# Recent Improvements to the NWChem COSMO Module

**DOI:** 10.1021/acs.jctc.5c01368

**Published:** 2025-11-05

**Authors:** Rafael de P. Soares, Daniel Mejía-Rodriguez, Edoardo Aprà

**Affiliations:** † Virtual Laboratory for Properties Prediction, 28124 Federal University of Rio Grande Do Sul, Porto Alegre 90035007, Brazil; ‡ Physical Sciences Division, 6865 Pacific Northwest National Laboratory, Richland, Washington 99352, United States; § Environmental Molecular Sciences Laboratory, 6865 Pacific Northwest National Laboratory, Richland, Washington 99352, United States

## Abstract

This work presents recent developments in the COSMO solvation model implementation in NWChem. A new cavity construction approach, based on the solvent-excluding surface (SES) and utilizing the well-established GEPOL algorithm, has been introduced. Additionally, a straightforward procedure to merge surface segments that are too close—often a source of numerical artifacts—has been implemented. The available methods for correcting outlying surface charges have also been reviewed and improved. To validate the new implementation, we computed dielectric solvation energies for a chemically diverse set of approximately 100 molecules, including neutral species, small ions, and common ionic liquid components. Results were compared to those from GAMESS using the double-cavity method as a reference. Although the double-cavity approach can be regarded as more accurate, the simpler correction schemes available in NWChem—based on scaling factors or Lagrange multipliers—can achieve excellent agreement if the potential is also properly corrected, with mean unsigned deviations of around 0.15 kcal/mol. Predictions of typical vapor–liquid and liquid–liquid equilibria using a COSMO-SAC variant based on NWChem also showed very promising results.

## Graphical abstract

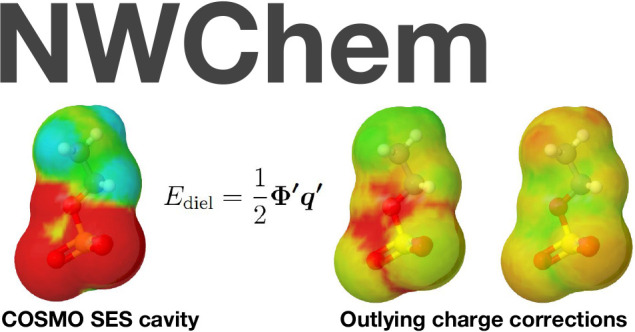

## 1 Introduction

NWChem (NorthWest Chemistry) is an open-source computational chemistry package that integrates a wide array of electronic structure and molecular simulation methods. Developed collaboratively by experts in theoretical chemistry, applied mathematics, and computer science, NWChem serves as a flexible and extensible platform for the implementation and testing of new theoretical approaches. As of this writing, the list of authors includes 107 contributors and continues to grow, highlighting the collaborative nature and ongoing development of the project. Distributed via GitHub under the Educational Community License version 2.0 (ECL 2.0), NWChem supports density functional theory (DFT) with both Gaussian and plane wave basis sets as well as Hartree–Fock (HF) and a range of post-HF correlated methods, including Møller–Plesset perturbation theory and coupled cluster techniques (single- and multireference). It also enables molecular dynamics simulations using quantum mechanical (QM), classical (MM), and hybrid QM/MM force fields. These features make NWChem a robust and widely adopted tool for tackling diverse scientific problems. More details on the code and its capabilities are available in recent perspective articles^1,2^ and the official repository at https://github.com/nwchemgit/nwchem.

NWChem has a broad user base and is widely used in academia and industry around the world.^2^ However, until recently, studies employing the conductor-like screening model (COSMO)^3^ implementation in NWChem have been relatively sparse in the literature.^4–8^ COSMO is a type of continuum solvation model. In this category of models, the solvent is treated as a dielectric, structureless medium coupled with a detailed quantum mechanical description of the solute. The solute charge distribution polarizes the continuum, which, in turn, polarizes the solute back. Excellent reviews summarizing the differences and similarities among quantum mechanical continuum solvation models are available in the literature.^9,10^ COSMO is now a widely adopted method for incorporating solvent effects and is available in a number of quantum chemistry packages.^1,11–20^ In addition to its general use for estimating solvation free energies and solvated geometries, significant interest in COSMO also stems from its quasi-chemical extension, COSMO-RS (Real Solvents)^21,22^ which enables the prediction of thermodynamic properties of general mixtures. Numerous studies have focused on improving COSMO-RS and its variants, such as COSMO-SAC^23,24^ including extensions that incorporate pressure effects in an equation-of-state framework.^25,26^ Moreover, surface charge densities computed by COSMO—or properties derived from them—have been employed to predict a variety of properties using Quantitative Structure–Property Relationships (QSPR) or machine learning approaches.^27,28^

Although free or open-source implementations of COSMO-SAC^29,30^ and COSMO-RS^31^ are currently available, they still depend on third-party software to perform the underlying COSMO calculations. In this regard, libraries implementing continuum solvation models such as COSMO are available—such as DDX^32^ and PCMSolver^33^—and have been interfaced with many quantum chemistry packages, including the open-source Psi4 package.^14^ It is worth pointing out that the polarizable continuum model (PCM) predates COSMO. Both are based on the definition of apparent surface charges on a cavity surface. In the original PCM method,^34^ the surface charges come from the normal component of the electric field. In a more recent version, IEF-PCM,^35^ the electric field is replaced by the electrostatic potential. COSMO also calculates apparent surface charges based on the electrostatic potential, but with an additional simplification: assuming the solvent is a conductor and then applying an empirical correction to the results.^9^ When this conductor simplification is employed in PCM codes, the variant is usually called C-PCM.^13^ Thus, both COSMO and C-PCM use identical matrices to get the apparent surface charges, but specific details may vary between implementations, including cavity construction, scaling in finite dielectric, and handling of outlying charges. In contrast, NWChem is, to the best of our knowledge, the only open-source quantum chemistry package that includes a built-in COSMO implementation with output ready to use by COSMO-RS/SAC codes, with different cavity construction methods and different methods for outlying charge correction. However, prior to this work, its functionality was limited in several respects. Cavity construction was restricted to the van der Waals type and not the expected *molecular* (solvent-excluding) surface.^36^ In addition, the available methods for outlying charge correction—which should address the small but non-negligible portion of the electron density that extends beyond the cavity boundary^37^—did not update the electrostatic potential used to compute the interaction energy.

In this work, we report two key improvements to the COSMO implementation in NWChem: (i) a new cavity construction scheme, including a workaround to address numerical issues that arise when surface segments end up positioned too closely; and (ii) a revised outlying charge correction approach that now properly adjusts the electrostatic potential. These developments have led to more consistent computations and substantially improved accuracy in solvation energy predictions. The enhancements described here have been incorporated into the official NWChem repository and are included in the 7.3.0 release.

## 2 Theory and Implementation

The fundamental idea behind COSMO, in contrast to other dielectric continuum models, is to approximate the dielectric medium by a scaled perfect conductor. This approximation enables the use of the relatively simple boundary condition of vanishing total potential instead of the more complex boundary conditions required by a general dielectric continuum. The solute is assumed to reside within a molecularly shaped cavity, which may be defined using van der Waals, solvent-accessible, or solvent-excluding surfaces. The procedure begins by discretizing the cavity surface into small segments. In the original method—referred to within NWChem as KS (after Klamt and Schüürmann^3^)—a uniform surface charge density σ_*i*_ is assumed for each segment *i*, resulting in a screening charge given by *q*_*i*_ = *s*_*i*_σ_*i*_, where *s*_*i*_ is the area of segment *i*. This is common to all *apparent surface charge* approaches.^38^ In our actual implementation, screening (point) charges *q*_*i*_ are placed in the center of the surface segments, as depicted in Figure 1.

**Figure 1:**
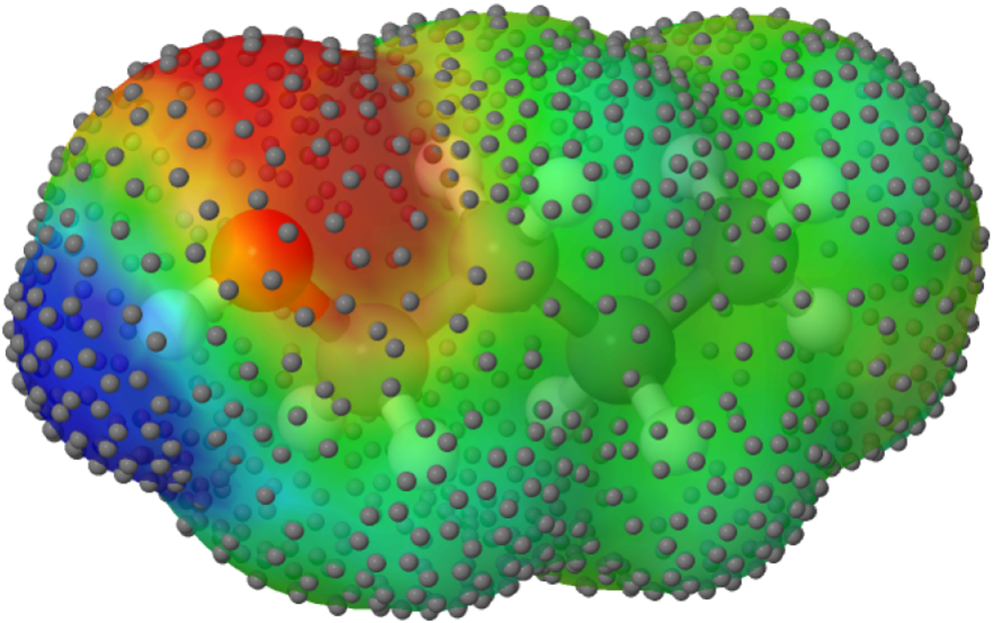
Solvent-excluding cavity for 1-butanol with colors given by COSMO charge densities σ_*i*_ calculated by NWChem. The color scheme is as follows: green represents a neutral charge, red saturates at 0.012 *e*/Å^2^, and blue saturates at −0.012 *e*/Å^2^. The small spheres represent the point charge positions at the center of surface segments.

In vector notation, the screening charges can be written as ***q*** = (*q*_1_, *q*_2_,..., *q*_*m*_), and the Coulomb interactions between these charges can be represented by a symmetric matrix ***A*** that depends only on the cavity geometry. The matrix ***A*** also takes into account the self-interaction of a homogeneous charge distribution by nonzero diagonal elements.^3^ If **Φ** denotes the vector of electrostatic potentials generated by the solute on each surface segment, the condition of vanishing total potential in a conductor leads to

1 0=Φ+Aq

where ***q*** is the vector of screening charges in a perfect conductor (ϵ = ∞).

Thus, the screening charges under the perfect conductor approximation can be obtained by solving the linear system of equations in eq 1. This is again similar to an earlier method known as the boundary element method (BEM) matrix inversion procedure.^38^ In NWChem, there are two available options for solving this system: the directive lineq 0 selects a dense matrix solver (default), while lineq 1 activates an iterative method. For large molecules, where the number of surface segments (and thus point charges) becomes significant (*m* > 7500), the code switches the default to the iterative method to improve performance.

In the YK variant (after York and Karplus^39^), surface elements are modeled using spherical Gaussian functions to eliminate the occasional Coulomb singularities (or near-singularities) present in the conventional point-charge model. The exponents are calibrated to reproduce the exact Born ion energy and to ensure a uniform surface charge density.

Both the KS and YK variants are available in NWChem and can be activated using the directives do_cosmo_ks and do_cosmo_yk, respectively. The COSMO approximation assumes that, at a finite dielectric constant ϵ, the screening charges should be scaled:

2 $$q_{\epsilon }=f_{\epsilon }q$$

where *f*_ϵ_ is a dielectric-dependent scaling factor.

Different options for the scaling factor are available in NWChem: screen ideal applies no scaling, *f*_ϵ_ = 1, and the dielectric constant is irrelevant, as the solvent is treated as a perfect conductor with ϵ = ∞; screen ks uses the original scaling by Klamt and Schüürmann,^3^ given by *f*_ϵ_ = (ϵ – 1)/(ϵ + 0.5); and screen st applies the modified scaling proposed by Stefanovich and Truong,^40^ defined as *f*_ϵ_ = (ϵ – 1)/ϵ.

### 2.1 New Solvent-Excluding Surface Cavity

The cavity is a fundamental concept in all continuum solvation models,^10^ although its shape and size may vary across different models and implementations. Ideally, the cavity should exclude the solvent while encompassing the majority of the solute’s charge distribution (this topic is discussed in more detail in subsection [Sec sec2.2]). It is widely accepted that the cavity should closely reproduce the molecular shape. Prior to this work, cavity construction in NWChem was limited to the van der Waals type—defined as a set of rigid, interlocking spheres of prescribed radii centered on each atom.

The construction of van der Waals (vdW) surfaces is relatively simple: each atomic sphere is first divided into many segments, and any segments that lie within other spheres are subsequently removed. However, molecular shapes are often irregular, and small regions that are inaccessible to solvent molecules are not uncommon. This intuitive observation underlies the concept of the solvent-excluding surface (SES).^10,36^ An SES, also referred to as the *molecular surface*,^36^ consists of two components: the contact surface and the reentrant surface. The contact surface corresponds to the portion of the vdW surface that is accessible to a probe sphere with a given radius. The reentrant surface is the inward-facing area of the probe sphere when it is in contact with more than one atom. As a simple example, Figure 2 shows the vdW and SES cavities for a particular conformation of *n*-nonane. As can be seen, the vdW surface exposes more area than the SES surface, since the SES accounts for the steric hindrance of solvent molecules.

**Figure 2:**
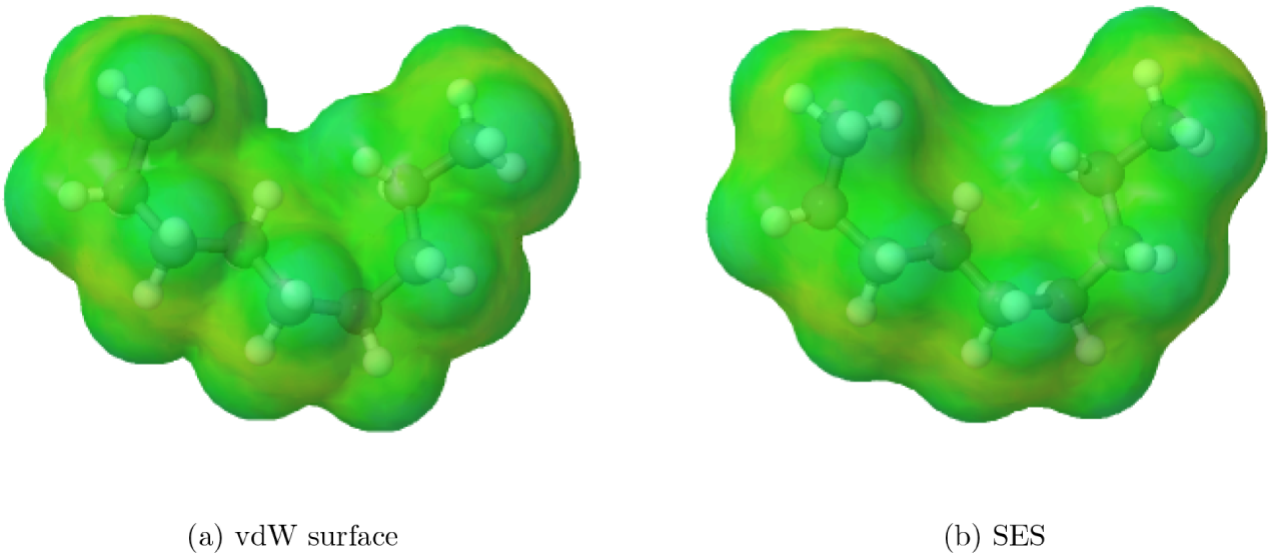
Different cavity surfaces for *n*-nonane in a particular conformation, chosen to highlight surface differences. The solvent-excluded surface (SES) was calculated using a probe radius of 1.3 Å.

Here, we report the addition of SES cavities in NWChem. The implementation is based on the well-known GEPOL93 algorithm.^36^ In GEPOL93, atomic spherical surfaces are initially divided into 60 triangular tesserae using a data-coded pentakisdodecahedron, which can be further refined by subdivisions in multiples of 4. In our implementation, users can increase the tessellation from 60 to 240 and 960 triangles by specifying ndiv coarse, ndiv fine (default), or ndiv xfine, respectively. It should be noted that the algorithm does not generate a true SES cavity but rather an approximate one by adding additional spheres to fill regions inaccessible to the solvent. Although this approximation can lead to an unphysical smoothing curvature, it has the advantage of not leaving large open areas as seen in some classical COSMO codes.^41^ The implemented method enables efficient calculation of not only molecular surface area and volume but also the coordinates of the surface tesserae.

In its standard implementation, after calculating the cavity’s total area and volume, GEPOL93 regroups the secondary triangles back into the original main triangles, resulting in a maximum of 60 tesserae per sphere. Since a finer tessellation might also be advantageous for the calculation of the COSMO surface charges, we modified the code to skip this final regrouping step.

Regardless of the SES cavity tessellation level (coarse, fine, or xfine)—and similarly for vdW cavities—the surface discretization can occasionally result in surface segment centers that are very close to one another in the vicinity of the boundary between spheres. In the point-charge treatment (KS method), if two point charges are placed too closely, a near-singularity in the Coulomb interaction may arise. This leads to numerical artifacts and instabilities during the matrix inversion required to compute the screening charges, occasionally resulting in convergence problems or abnormally large charge densities, as illustrated in Figure 3.

**Figure 3:**
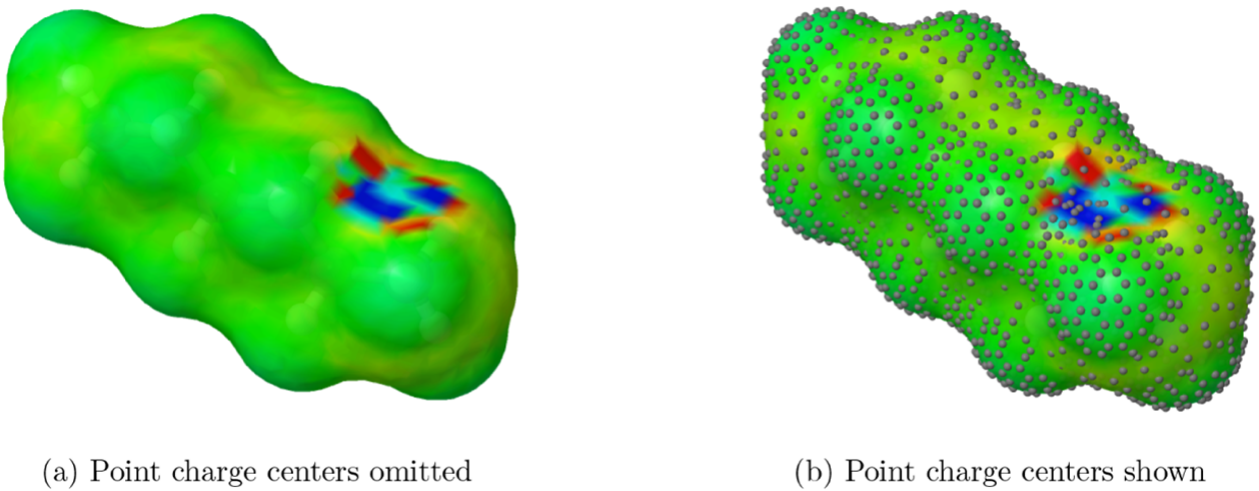
Abnormally large surface charge densities for *n*-hexane result from near singularities. As per the color scheme in Figure 1, these densities should all be near zero (green). The elevated values originate from surface segments that are excessively close and have different areas, with effects potentially extending beyond those specific segments.

This type of numerical issue has also been observed in other implementations, such as in ORCA.^13^ In the work of Müller et al.,^42^ the problem is mitigated by postprocessing and discarding surface segments whose area falls below a specified threshold (0.01 Å^2^). Although removing these small segments has a minimal effect on the total energy, it undermines the conductor boundary condition, which requires a vanishing electrostatic potential across the surface. Because a highly charged segment can influence the charge distribution on neighboring segments (as illustrated in Figure 3), this approach is not recommended. It should be noted that the YK method—based on Gaussian spherical charge distributions (smeared charges)—does not suffer from any of these numerical artifacts and that it is the default option when executing conductor-like calculations within ORCA and NWChem.

For now, the issue of near-singularities in the KS method is addressed by a simple approach in our implementation. More sophisticated schemes, such as the *variable area* method,^43^ can be implemented in future versions. First, the minimum expected distance between surface segments, *d*_min_, is computed based on the solvent probe radius (rsolv) and the selected tessellation level (ndiv). Next, any pair of surface segments with centers closer than *d*_min_ are merged by relocating them to their centroid and summing their surface areas. This merging procedure is applied during cavity construction, ensuring that the subsequent point charge calculation does not encounter near-singularities. To offer flexibility, users can adjust the merging behavior via the mfactor parameter, which scales to *d*_min_. The default value is mfactor 1.0, which has proven sufficient in all test cases conducted so far. The results shown in Figure 3 correspond to mfactor 0.0, i.e., with segment merging disabled. With the YK method, segment merging is not necessary.

### 2.2 Updated Outlying Charge Treatment

All methods that exploit cavities and boundaries to separate solute and solvent domains, are subject to *outlying charges* when quantum mechanical charge distributions are used to describe the solute.^10^ This is due to the fact that a portion of the electronic charge of the solute always lies outside any cavity of reasonable size. Typically, cavities are constructed using atom radii slightly larger than van der Waals radii, and only about 1% of the total electron density resides outside the cavity.^44^ The COSMO method inherently reduces errors due to outlying charges by nearly an order of magnitude compared with other dielectric methods. However, the residual screening charge defect may still be significant, especially for anions.^44^ Errors are typically below 1 kcal/mol for neutral compounds but can be significantly larger for anions.^19^

When the outlying charge correction is accomplished by scaling factors (directive charge_correction scale), the NWChem implementation splits the solute potential and the resulting screening charges into nuclear (***q***_*N*_) and electronic (***q***_*e*_) contributions, which is a common approach in continuum models.^38^ The linear system of equations in eq 1 is also solved separately with **Φ**_*N*_ and **Φ**_*e*_, and the total point charges are given by ***q*** = ***q***_*N*_ + ***q***_*e*_.

With separate nuclear and electronic components, the *charge rule* ^45^ requires that the following relations hold,^46^

3 $$-Q_{N}=1^{T}q_{N},,-Q_{e}=1^{T}q_{e}$$

where *Q*_*N*_ and *Q*_*e*_ are the total nuclear and electronic charges of the solute.

These relations are not exactly satisfied in actual calculations for two main reasons. First, this is due to numerical inaccuracies arising from the discretization of the cavity surface. Second, it is because of the presence of outlying charge, which should affect ***q***_*e*_ exclusively,^46^ although in neutral molecules there should be exactly as much *net* charge outside the cavity as inside.^47^ Nevertheless, two scaling factors are used to enforce eq 3: *f*_*N*_ = *Q*_*N*_/ (**1** ^T^ ***q***_*N*_) and *f*_*e*_ = *Q*_*e*_/ (**1** ^T^ ***q***_*e*_). These factors are then used to compute corrected point charges: $q_{N}^{\prime }=f_{N}q_{N}$ and $q_{e}^{\prime }=f_{e}q_{e}$ . The nuclear factor *f*_*N*_ generally is near unity, suggesting that it could potentially be disregarded. From a theoretical standpoint, since all nuclear charges reside inside the cavity, *f*_*N*_ should be unity for perfect surface discretization.

While this approach effectively corrects the global error in the screening charges, it still leaves residual errors in the electrostatic potential. To address this, we additionally correct the total potential to enforce the boundary condition for the corrected charges:^44^

4 $$\Phi^{\prime }=-A\left(q_{N}^{\prime }+q_{e}^{\prime }\right)=-Aq^{\prime }$$

Finally, since the nuclear potential should remain unaffected by outlying electronic charges, the corrected electronic potential is obtained by subtracting the nuclear contribution:

5 $$\Phi_{e}^{\prime }=\Phi^{\prime }-\Phi_{N}$$

The do_cosmo_file command also outputs the values of *f*_*N*_, *f*_*e*_, and the corrected point charges (***q*** ′), as well as the coordinates of the segment centers and additional related information.

An alternative method available in NWChem uses Lagrange multipliers to enforce Gauss’s law and determine corrected charges. This approach is activated by the charge_correction lagrangian directive. The core idea is to derive the surface charges by minimizing an energy functional rather than solving the linear system in eq 1. From an optimization perspective, a Lagrange term is added to enforce the total charge constraint. For this particular case, the solution can again be reduced to a linear problem and yields corrected surface charges ***q*** ′. Further details can be found in the original work by York and Karplus.^39^ As before, we propose here to correct the total potential **Φ**′ using the corrected charges ***q*** ′, as shown in eq 4.

Regardless of the method, all energy calculations should then use the corrected potentials (**Φ**′, $\Phi_{e}^{\prime }$ ) and the corrected point charges (***q*** ′, $q_{e}^{\prime }$ ). For instance, the *dielectric energy* should be calculated by^37,44^

6 $$E_{\mathrm{diel}}=\frac{1}{2}\Phi^{\prime }q^{\prime }$$

Prior to this work, only the point charges were corrected, while the potentials remained unmodified. For comparison purposes, this legacy behavior can be restored by using the potcorr directive.

## 3 Computational Details

All calculations reported in this work were performed using locally modified versions of either NWChem^2^—with enhancements already merged into the master branch of the official NWChem repository and included in the 7.3.0 release—or GAMESS,^12^ modified as previously described.^48,49^ We employ the widely used hybrid functional B3LYP^50,51^ in combination with the def2-SVPD basis set.^52^ All geometries were optimized in the gas phase^53,54^ and are available at https://github.com/lvpp/sigma,^48,49^ followed by single-point COSMO calculations using the KS method and SES cavities. Other combinations of methods (YK or KS) and cavity types (vdW or SES) are possible but were not investigated in this work. Instead of using custom-optimized atomic radii,^22^ we employed previously tabulated values,^55^ uniformly increased by 18%. As is customary, the solvent probe radius was set to 1.3 Å. In the case of SES cavities, this probe radius does not add thickness but is used to determine only the region accessible to the probe sphere.

The input file shown in Scheme 1 illustrates a typical single-point COSMO calculation using the B3LYP functional and the def2-SVPD basis set within NWChem. The geometry of the water molecule is specified, followed by the basis set definition, DFT settings, and COSMO-specific directives. This setup includes ideal conductor screening, employs the KS method with an SES cavity, and applies outlying charge correction via scaling. The directives do_cosmo_ks, cavity ses, and charge_correction scale override the default settings in NWChem—the YK method with a vdW cavity and Lagrangian outlying charge corrections—which are retained to preserve backward compatibility.

**Scheme 1:**
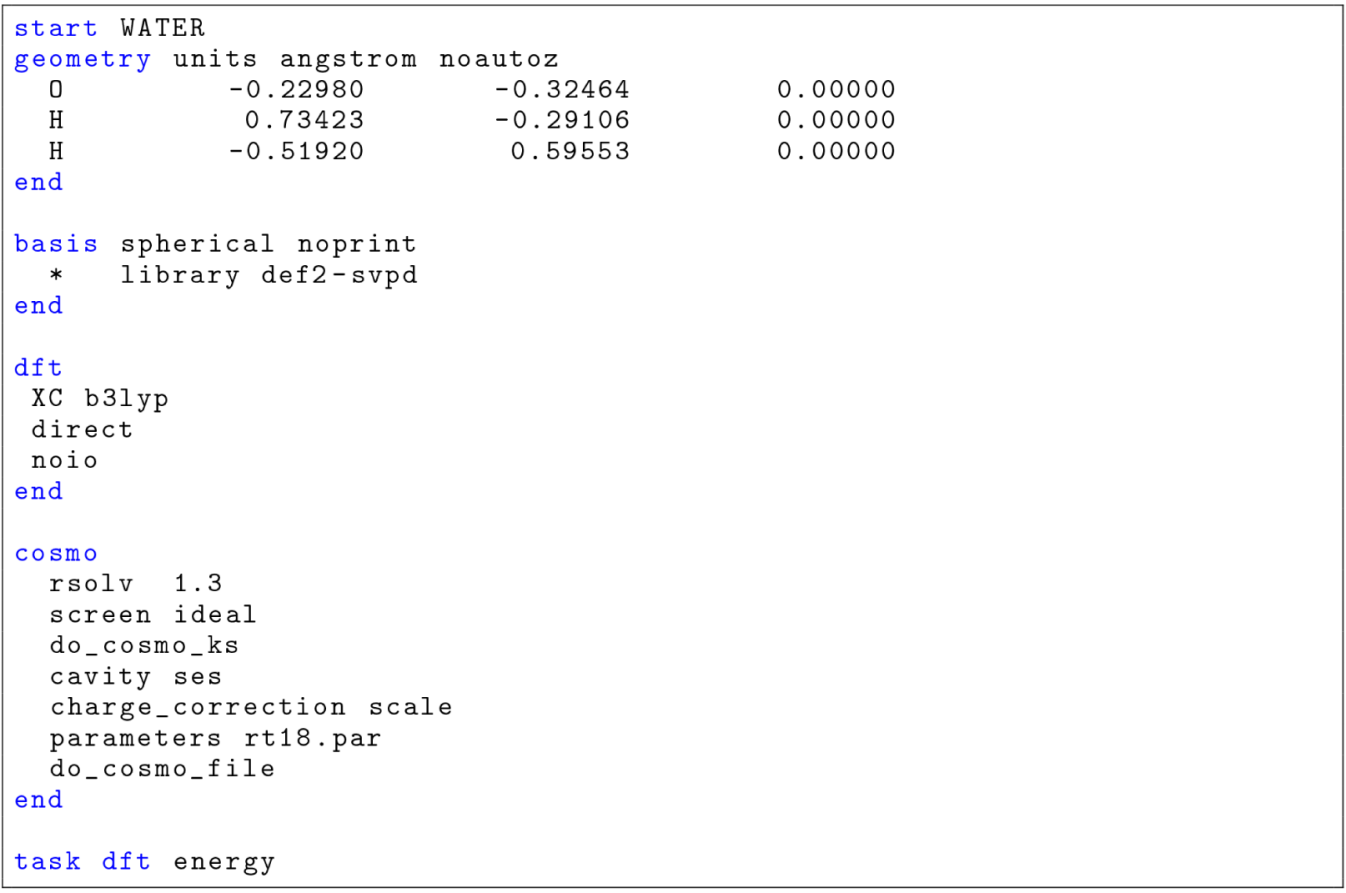
Input File for Single-Point COSMO Calculation for Water in NWChem

Atomic radii, increased by 18% relative to the tabulated values by Rowland and Taylor,^55^ are provided in a separate file named rt18.par. The do_cosmo_file directive instructs NWChem to output detailed COSMO data, including corrected charges and electrostatic potentials.

The output shown in Scheme 2 illustrates a typical COSMO file generated when the do_cosmo_file directive is included. The format is similar to the one generated by Turbomole,^20^ allowing for easier adaptation in COSMO-based workflows. It contains the relevant COSMO configuration settings along with various related data. Total gas, solvated, and dielectric energies are listed. The file also includes the molecular geometry, as well as the coordinates of surface segment centers, their associated area, charge, charge densities, and solute potential.

**Scheme 2:**
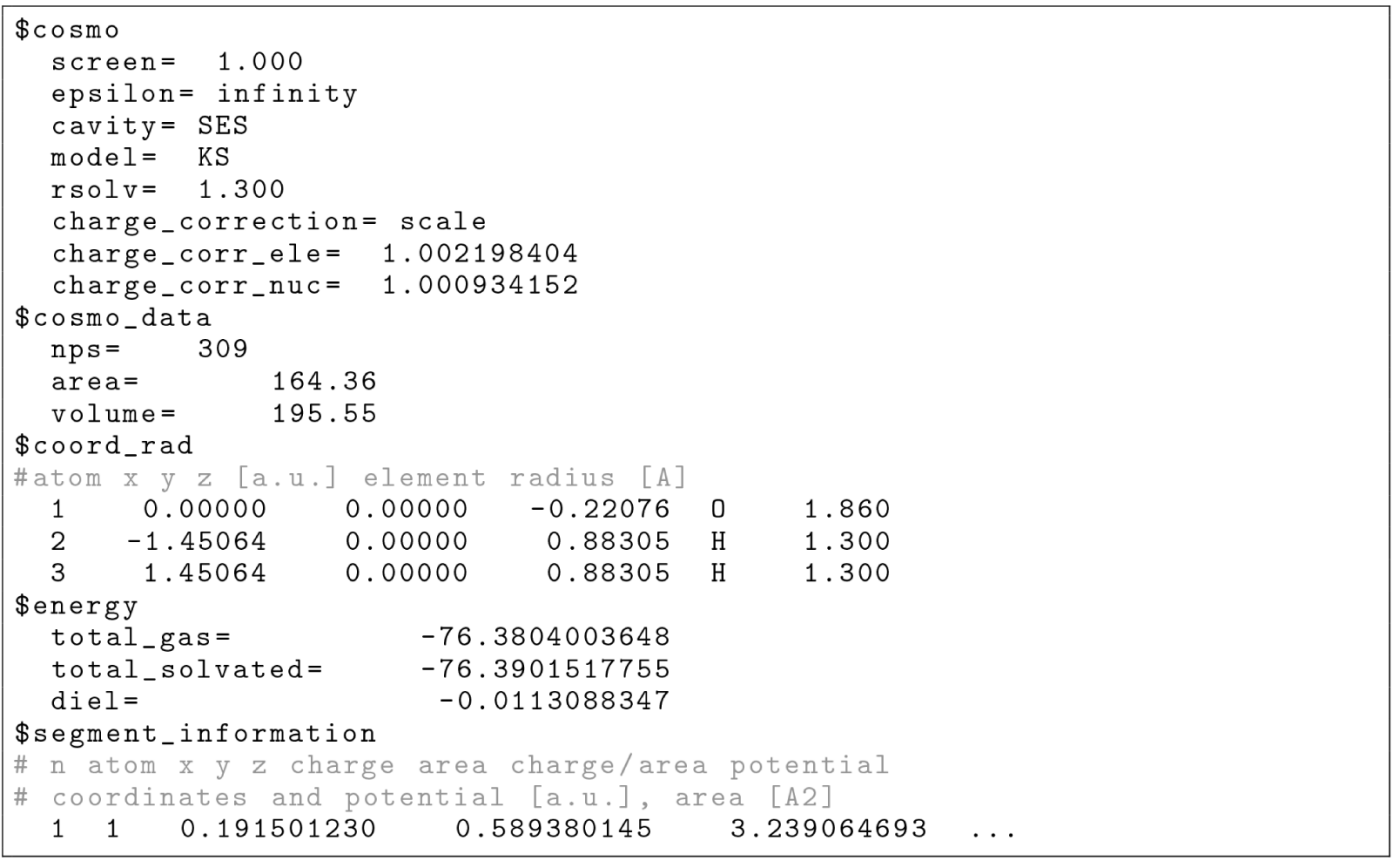
Excerpt from the NWChem COSMO Output File for Water, Showing the Initial and Most Relevant Portion

Comparisons among implementations were restricted to the ideal screening case (ϵ = ∞). For this purpose, we assembled a chemically diverse set of approximately 100 molecules, including neutral compounds, common cations and anions, as well as representative ions typically found in ionic liquids. Detailed results for computed properties are provided in the Supporting Information. Among the available methods in GAMESS, the outlying charge correction was performed using the double-cavity (DC) approach.^37^ All molecular surfaces shown in the figures, as well as the σ-profiles and phase equilibrium predictions for mixtures, were generated using JCOSMO.^24,29,48,49^ This tool is capable of reading COSMO files produced by various quantum chemistry packages, including NWChem, GAMESS, and Turbomole. For phase equilibrium predictions, COSMO computations use an SES cavity with the KS method and the scaled outlying charge correction.

## 4 Results and Discussion

### 4.1 SES Consistency

To assess the consistency of our new SES cavity generation code, we compared its results with the cavity construction available in GAMESS, which is also of the SES type. As a first step, we computed total cavity surface areas and volumes using both methods for a chemically diverse molecular data set (individual results available in the Supporting Information). As shown in the parity plots in Figure 4, the agreement between the two implementations is excellent, with coefficients of determination (*R* ^2^) exceeding 0.995 for both properties. These results confirm that the SES implementation in NWChem yields cavity geometries that are highly consistent with those generated by GAMESS. Visual inspection of all generated surfaces and their associated segment centers (as shown in Figure 3) revealed no anomalies or artifacts. In addition, an automated check of all segment charge densities confirmed that no unusually high values were present.

**Figure 4:**
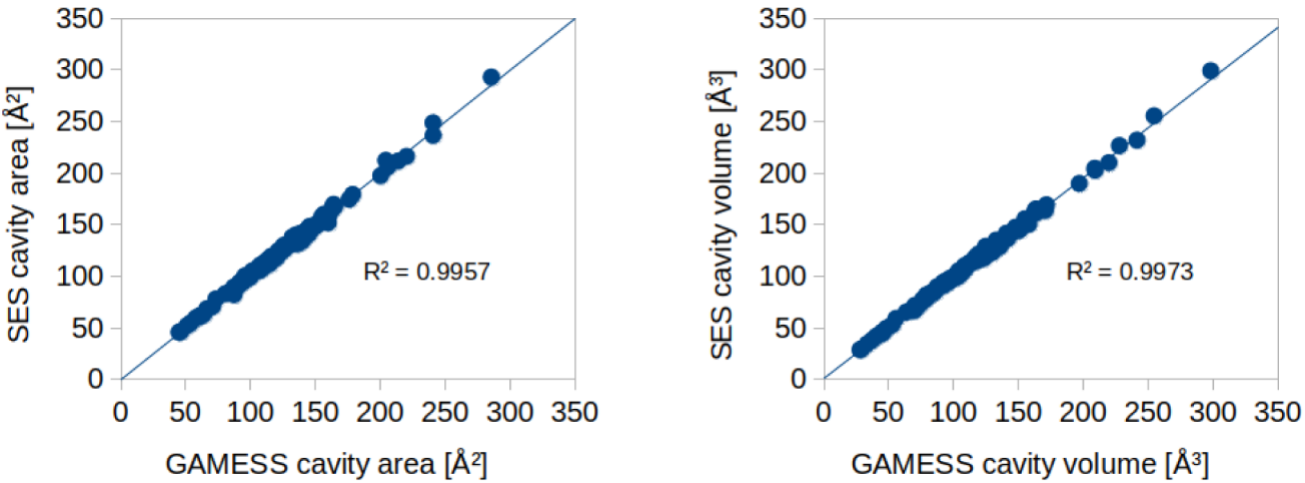
Parity plots comparing the total cavity surface area and volume from the new SES implementation in NWChem to those from the default GAMESS COSMO cavity construction. The straight line in each plot represents a linear fit to the data.

### 4.2 Outlying Charges and Dielectric Energy

Next, we evaluated the total charge defect—defined as the sum of the screening charges and the total charge of the solute—for both NWChem and GAMESS. In GAMESS, the double-cavity (DC) method was employed to mitigate outlying charges. It should be noted that all available methods rely on heuristic approximations. In the absence of a fully rigorous alternative, the energies calculated with the DC method were taken as our benchmark. Figure 5 presents the charge defect results for our dataset using GAMESS and NWChem without any correction (directive charge_correction off).

**Figure 5:**
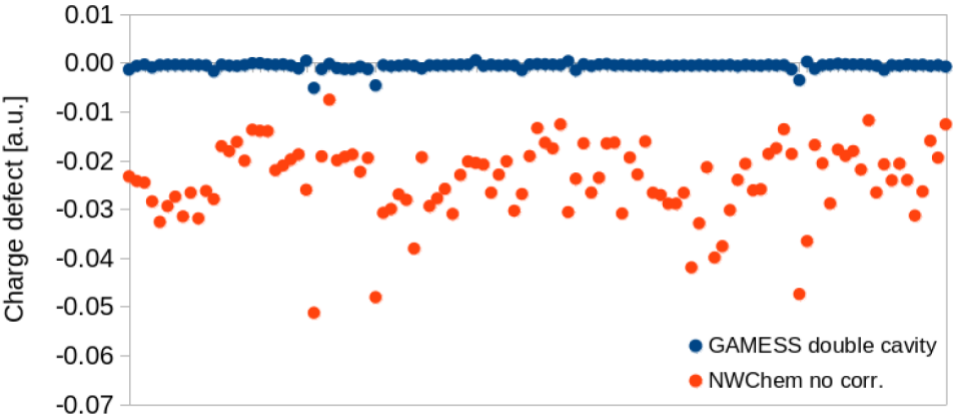
Charge defects for GAMESS (using the double-cavity method) and NWChem without charge correction. NWChem results with charge correction are omitted, as the corresponding defects are on the order of 1 × 10^–9^ or smaller.

As previously reported,^44^ the charge defect can be significant if no correction is applied. The double-cavity method achieves a very good correction of outlying charge defects, though small residuals on the order of −0.01 *e* still remain for the investigated data set. In contrast, NWChem’s charge correction approaches—whether using scaling factors or Lagrange multipliers—consistently reduce the defect to numerical precision (on the order of 10^–9^ or smaller), which are not shown in Figure 5 as they would be visually negligible. Such small residuals are expected: in the scaling approach, the defect is exactly redistributed, while in the Lagrange method, it is minimized as part of the surface charges solution. Although the global error is near zero, these corrections may yield local errors, since neither method is actually rigorous.^45,47,56^

Thus, we also evaluated the dielectric solvation energy (see eq 6) for all molecules in the data set. As shown in Figure 6, both charge correction methods in NWChem yield results that align almost perfectly with those from the GAMESS double-cavity benchmark, in addition to eliminating global charge defects. Without any correction, the mean unsigned deviation is approximately 0.6 kcal/mol, with maximum deviations reaching nearly 10 kcal/mol for anions. When applying either correction method, deviations are significantly reduced, with the largest below 1 kcal/mol and the average around 0.15 kcal/mol. It is important to emphasize that such low deviations are only achieved when the electrostatic potential—along with the screening charges—is corrected (eq 4); with the previous implementation, deviations still exceeded 4 kcal/mol. As before, detailed numerical results are provided in the Supporting Information.

**Figure 6:**
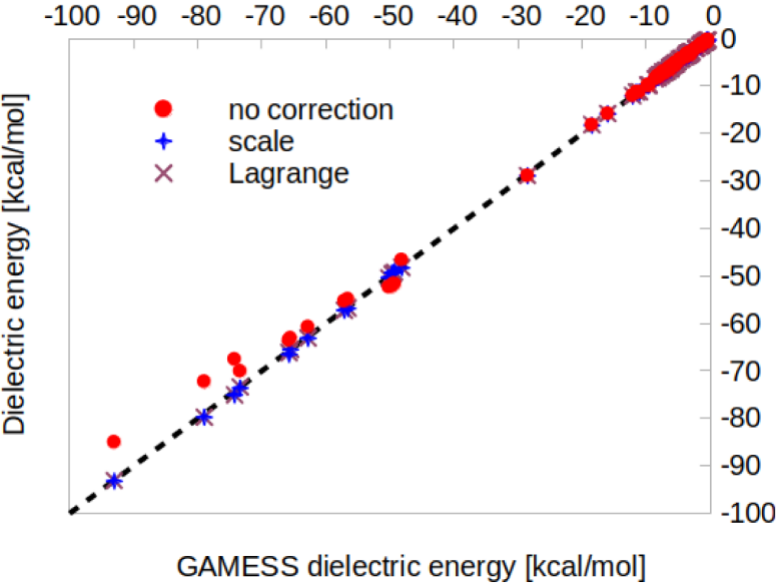
Dielectric solvation energies from GAMESS (using the double-cavity method) and NWChem, without any correction and with either a scaling factor or Lagrange multiplier charge correction, limited to −100 kcal/mol for better visualization. The dashed line represents perfect agreement *y* = *x*.

### 4.3 Limitations of Charge Correction Methods

The findings from the previous section indicate that the precise spatial distribution of surface charge corrections has little impact on the total dielectric energy. However, the local corrections can become relevant in the COSMO-RS/SAC calculations. We examine some specific cases in more detail.

For the cases in our database, the largest deviation between the double-cavity and NWChem methods occurs for Br^–^, followed by Cl^–^. For spherically symmetric anions, all corrections should produce the same result, and the observed difference actually arises from limitations in the double-cavity implementation. In these cases, the double-cavity method still leaves approximately 0.005*e* outside the cavity. In both GAMESS and Turbomole, the outer cavity used for the outlying charge correction is generated by projecting the surface outward by 85% of the solvent radius. For spherically symmetric systems, increasing the solvent radius effectively enlarges the outer cavity without the introduction of other effects. We tested the sensitivity of the results by doubling the solvent radius. This decreased the dielectric energy, bringing it closer to the values obtained from NWChem.

For nonspherical molecules, Klamt and Jonas^44^ argue that the double-cavity method is more rigorous. However, its practical implementation requires a correspondence between the inner and outer cavity surface segments. As a consequence, current implementations project only the contact spherical portions of the surface to construct the outer cavity, leaving the reentrant portion uncorrected. This approximation inevitably results in a coarser outer cavity, which may even be partially open or lead to the numerical artifacts previously discussed (see Figure 3). Apart from these numerical issues, the procedure can produce rather arbitrary changes in the dipole and higher multipole properties of the surface charge distribution and has already been criticized for being unphysical.^45,47^

In Figure 7, the surface charge densities for acetonitrile, as well as the corrections $\Delta \sigma_{i}=\sigma_{i}^{\prime }-\sigma_{i}=\left(q_{i}^{\prime }-q_{i}\right)/s_{i}$ , are shown. Interestingly, in this case, the scaling method (that separates electronic and nuclear contributions) successfully captures an intuitive correlation between the total screening charges and outlying charge corrections. Hydrogens are largely unaffected by the scaling approach, while the Lagrangian method fails to capture this correlation. Corrections from the double-cavity method appear rather irregular; most of the green regions are left uncorrected simply because they do not belong to the spherical portion of the surface—for which we explicitly display the segment centers. Surprisingly, the anticipated correlation with the total charge density^44^ is not observed in this case. We note that these corrections are quite small, necessitating a much finer color scale (with red saturation at 0.0002 *e*/Å^2^) in order to visualize them.

**Figure 7:**
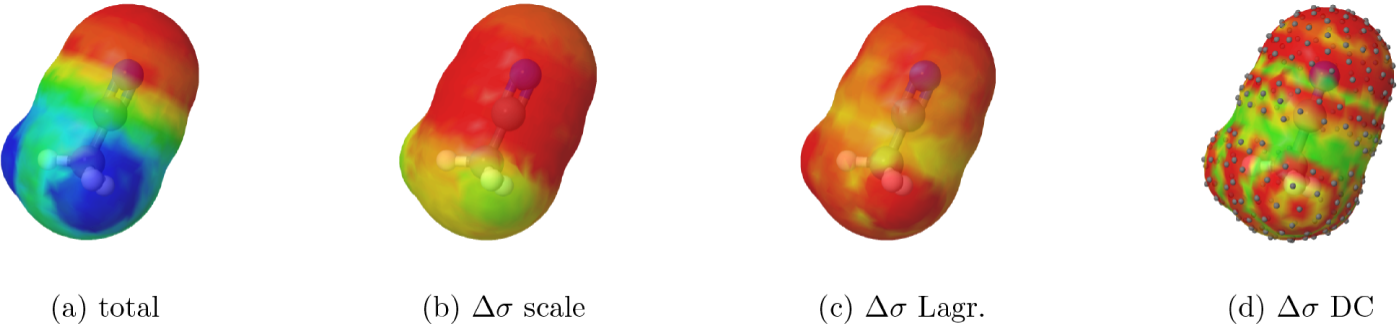
Surface charge densities for acetonitrile and corrections with different methods. For total density (a), red saturates at 0.01 *e*/Å^2^. For corrections (b-d), red saturates at 0.0002 e/Å^2^. For the double-cavity (DC) (d), we explicitly display the segments where the corrections were applied.

The case of 1,2-dichloroethane is illustrated in Figure 8. Once again, the scaling method avoids assigning charge corrections to hydrogen atoms, in contrast to the Lagrange method. The corrections from the double-cavity approach now correlate with the screening charges but exhibit some numerical artifacts, including unexpectedly small negative corrections over hydrogens. As before, a significant fraction of surface segments remains uncorrected in the double-cavity method since they do not lie on spherical portions of the cavity.

**Figure 8:**
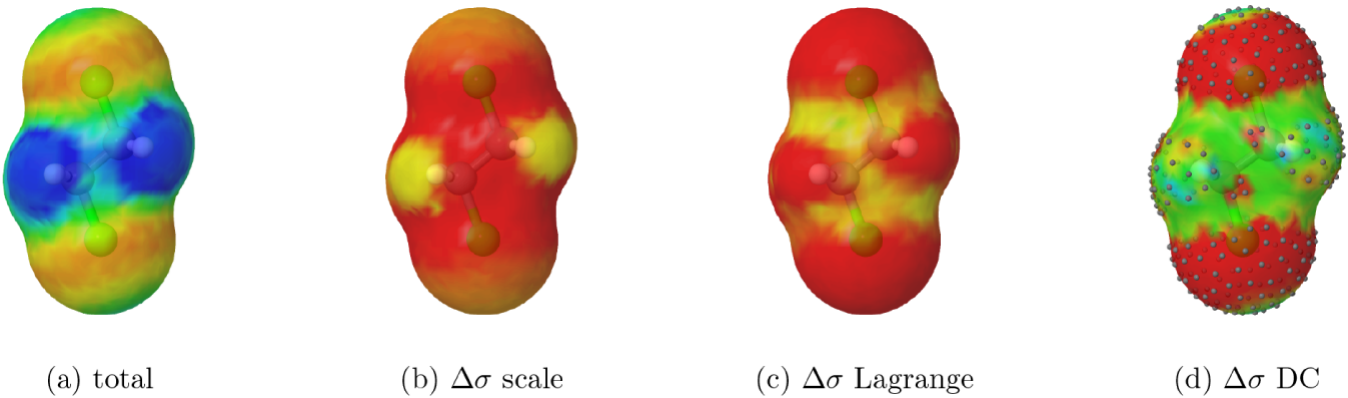
Surface charge densities for 1,2-dichloroethane and corrections with different methods. The color and segment convention is as in Figure 7.

As a final example, the nonspherical ethyl sulfate anion is shown in Figure 9. Since charge corrections tend to be more pronounced for anions, the correction images now use a different color scale, with red saturating at 0.0004 *e*/Å^2^. As in previous cases, the scaling method avoids assigning corrections to hydrogen atoms. However, it does not fully concentrate the corrections in the most negatively charged regions. Once again, the double-cavity method exhibits some numerical artifacts. While it is able to focus corrections in the more negatively charged areas, it also assigns corrections to the terminal methyl group—an outcome that is counterintuitive.

**Figure 9:**
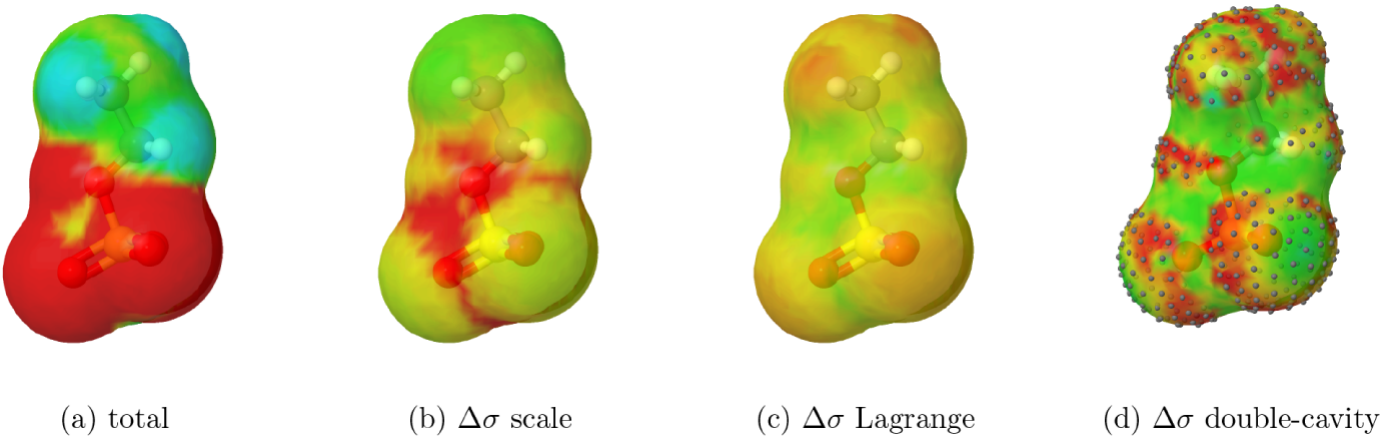
Surface charge densities for the ethyl sulfate anion and corrections by different methods. The color and segments are consistent with Figure 7, but for (b-d), red saturates at 0.0004 *e*/Å^2^.

All methods appear to alter the dipole and higher multipole characteristics of the surface charge distribution, with particular emphasis on the tested double-cavity implementation. Implementing approaches that correct the outlying charge effect and satisfy the *charge rule* without affecting the higher multipole moments—as proposed by Chipman^45,47^ —or developing improved double-cavity methods remains a worthwhile objective.

### 4.4 Prediction of Mixture Fluid Phase Equilibria

In this section, we present a few illustrative results on phase equilibrium predictions based on the COSMO computations performed with NWChem. The sources of experimental data are indicated in the figure captions. A COSMO-SAC variant was calibrated within JCOSMO (hereafter referred to as CS25) for use with NWChem SES cavities. As is customary in the literature^48,49^ universal parameters must be recalibrated when the underlying COSMO method is modified. The calibration was based mostly on binary infinite dilution activity coefficient data collected in previous works^24,57^ details of this calibration will be published elsewhere.

Figure 10 presents typical examples of positive and negative deviations from Raoult’s law. In the acetone/cyclohexane mixture, positive deviations are observed due to the polar–apolar nature of the components, as evidenced by the surface charge density histograms (σ-profiles) in Figure 13. For the chloroform/diethyl ether mixture, chloroform acts as a hydrogen-bond (HB) donor, while the ether serves solely as an acceptor—this distinction is also clearly reflected in the σ-profiles in Figure 13. In both cases, the phase equilibrium predictions are nearly perfect.

**Figure 10:**
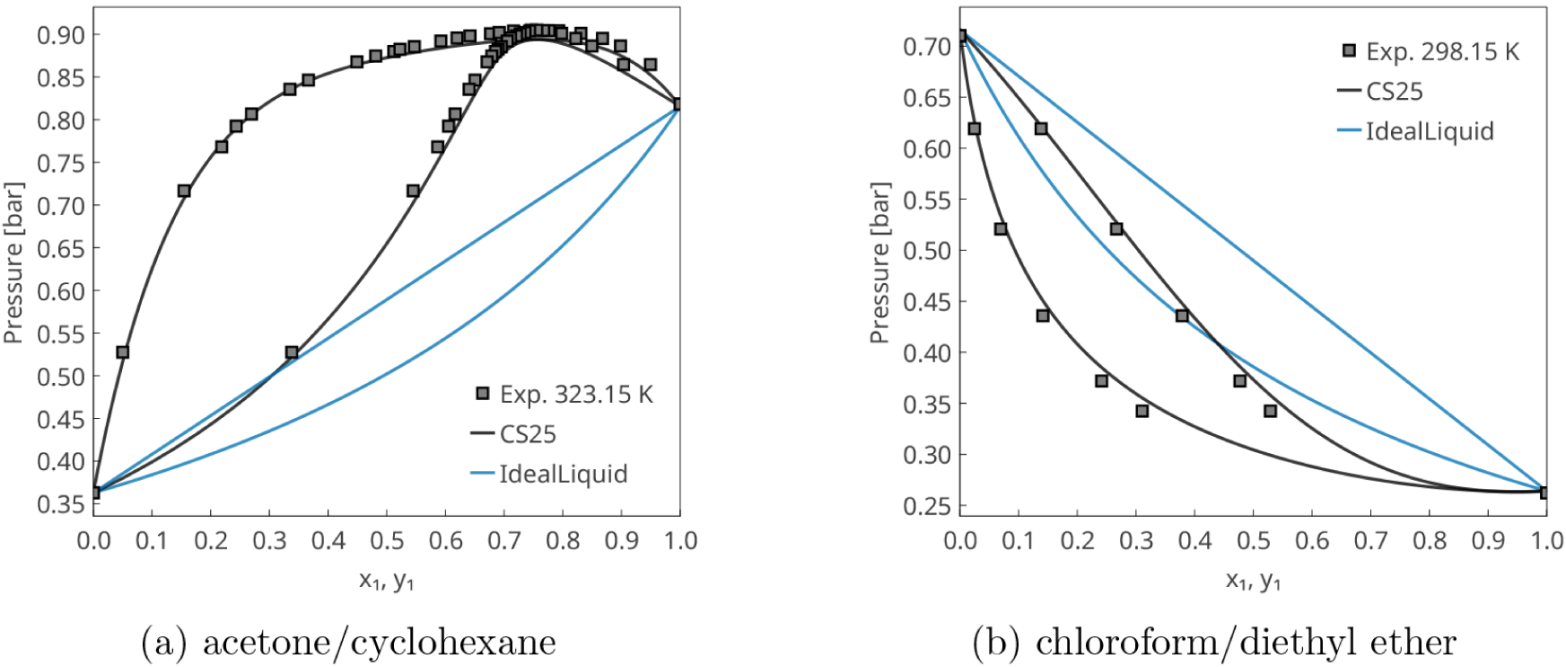
Vapor–liquid equilibrium predictions with a modified COSMO-SAC model based on NWChem (CS25), ideal liquid (Raoult’s law) predictions, and experimental data from the literature^58,59^ show cases with excellent agreement with experimental data.

In Figure 11, VLE predictions with less favorable agreement with experimental data are presented. These results illustrate the typical challenges in describing mixtures containing carboxylic acids without an explicit treatment of dimerization. A similar difficulty arises for mixtures of amines with HB donors, as exemplified by the ethanol/triethylamine system. In both cases, the model correctly predicts the positive deviation from Raoult’s law, although the magnitude is inaccurate.

**Figure 11:**
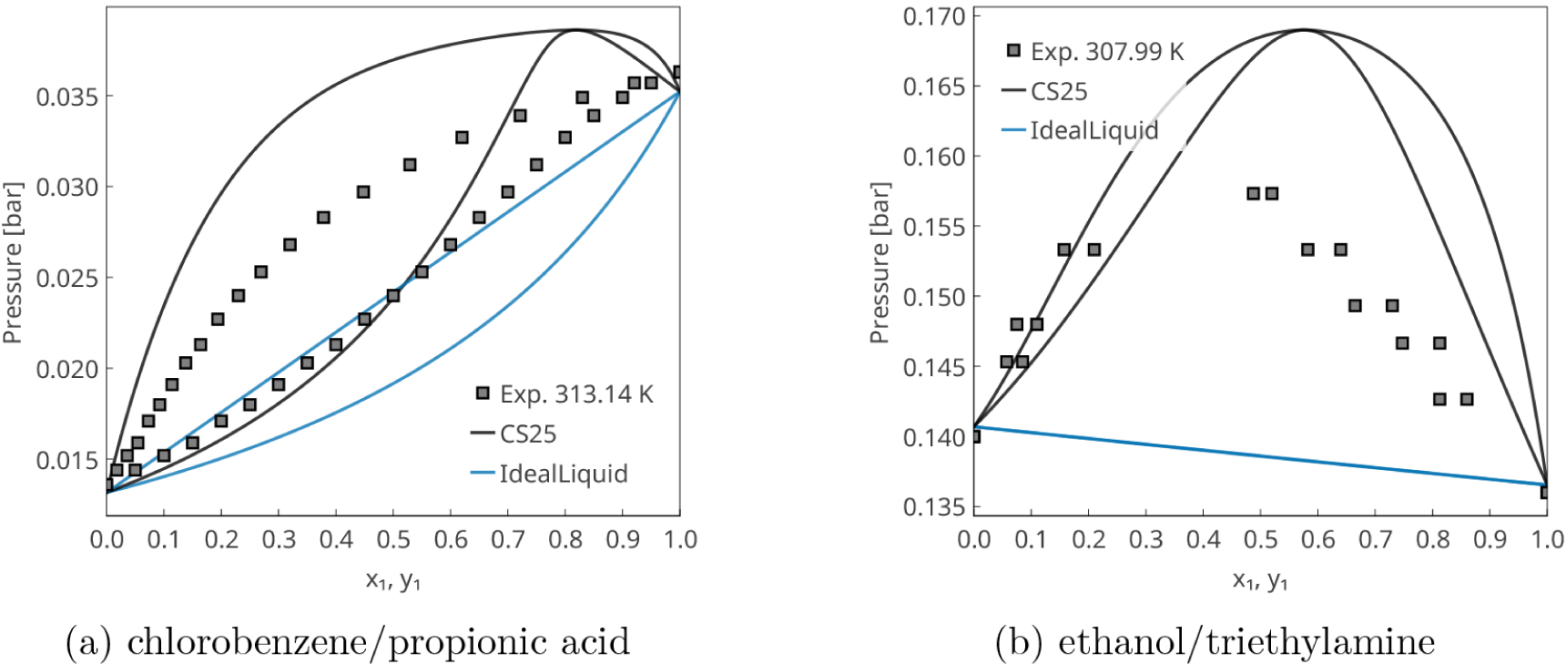
Vapor–liquid equilibrium predictions with a modified COSMO-SAC model based on NWChem (CS25), ideal liquid (Raoult’s law) predictions, and experimental data from NIST TDE^60^ show cases with less favorable agreement with experimental data.

Figure 12 shows two examples involving liquid–liquid phase separation. In both cases, the solubility of the organic solvent in the aqueous phase is lower than that of water in the organic phase. This asymmetry is accurately captured in the model. Additionally, the mutual solubility of the *n*-pentanol/water system is higher than that of the 2-pentanone/water system, which is also well-predicted. As usual, σ-profiles help interpret and explain these results; Figure 13 also includes the profiles for these substances.

**Figure 12:**
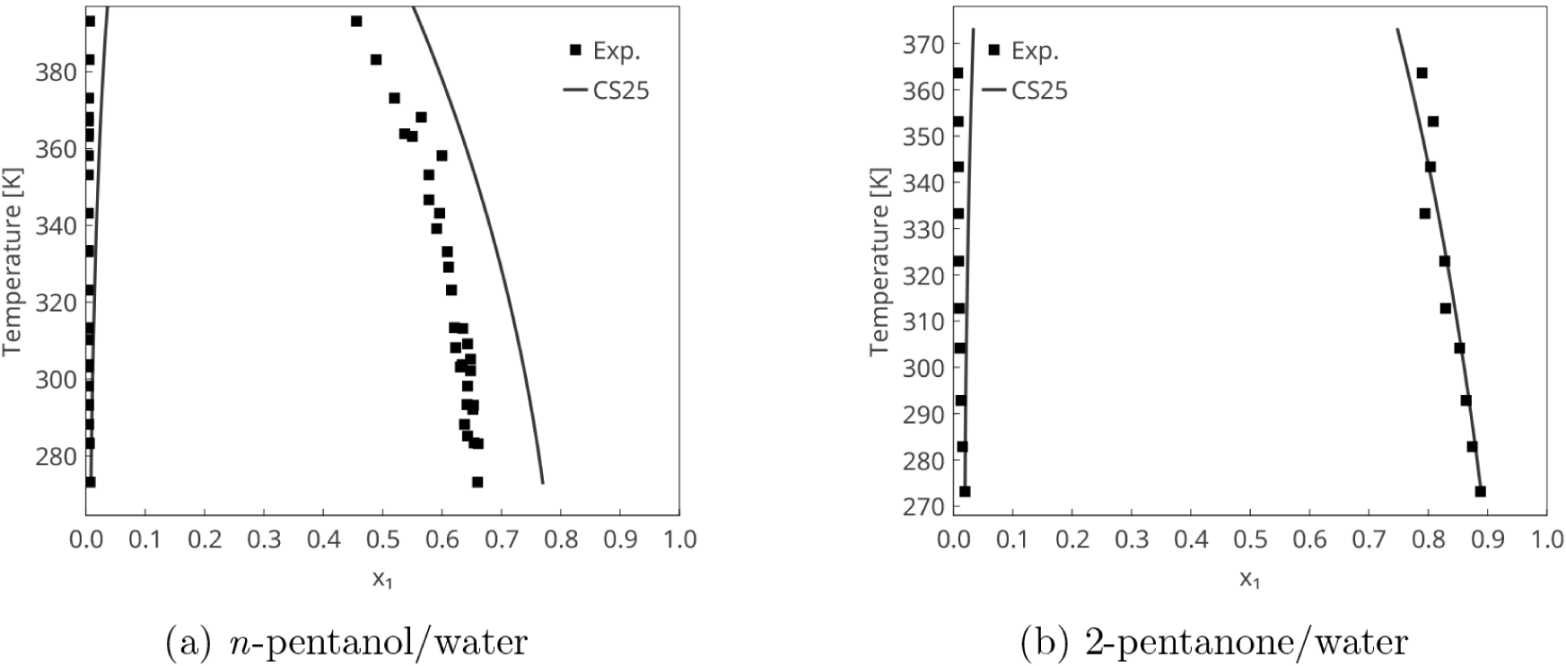
Liquid–liquid equilibrium predictions using a modified COSMO-SAC model based on NWChem (CS25) compared with experimental data from the literature.^61,62^

**Figure 13:**
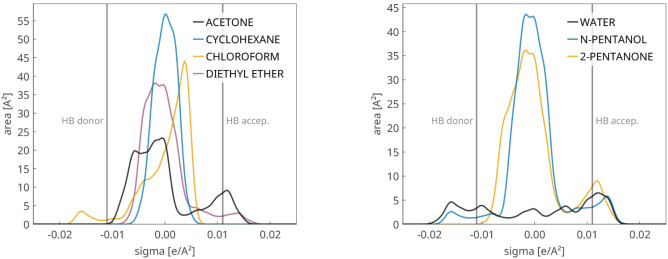
Surface charge density histograms (σ-profiles) for some of the substances used in the previous phase equilibrium examples, indicating the cutoffs for hydrogen-bond (HB) donor and acceptor regions.

## 5 Conclusions

In this work, we report the implementation of solvent-excluding surface (SES) generation in NWChem, based on the well-established GEPOL93 algorithm. A classical challenge in COSMO models, when based on point charges—the appearance of near-singularities—was addressed through a straightforward segment-merging strategy that combines surface elements positioned too closely. More advanced schemes may be explored in future developments.

Several outlying charge correction methods were investigated. The scaling and Lagrange multiplier-based approaches implemented in NWChem were reviewed and compared to the double-cavity method available in GAMESS. After the correct treatment for the electrostatic potential was implemented in NWChem, all charge correction methods produced similar dielectric solvation energies. However, they differed in how the corrections were spatially distributed over the molecular surface. Notably, all of the methods appear to introduce significant and somewhat arbitrary changes to the dipole and higher multipole moments of the surface charge distribution. Developing correction schemes that eliminate outlying charges without perturbing these higher-order moments remains an important direction for future research.

Finally, NWChem is now capable of generating COSMO surface charge density distributions (σ-profiles) using SES cavities. These profiles have been successfully combined with COSMO-SAC to predict various types of phase equilibria.

## Supplementary Material



## Notes

Detailed results organized by chemical species (XLSX)
